# PReS-FINAL-2250: Presentation of tuberculosis in patients using anti-TNFS drugs: report of 3 cases

**DOI:** 10.1186/1546-0096-11-S2-P240

**Published:** 2013-12-05

**Authors:** BE Bica, LM Saldarriaga Rivera, H Tupinambá, MNL Azevedo

**Affiliations:** 1 Rheumatology, Universidade Federal do Rio de Janeiro, Rio de Janeiro, Brazil

## Introduction

Inhibitors of tumor necrosis factor (iTNF) are drugs used to control chronic inflammatory arthritis in cases that are refractory to treatment with DMARDS. TNF is critical in preventing Mycobacterium tuberculosis reactivation; thereby decreased TNF-α activity suggests that the cytokine has a key role in the control of latent tuberculosis.

## Objectives

To describe the presentation of Tuberculosis (TB) in 3 patients using iTNF treated at the Rheumatology Department of the Hospital Universitário Clementino Fraga Filho, Universidade Federal do Rio de Janeiro (HUCFF-UFRJ).

## Methods: Case Report

Case 1: Female patient, 20 yo, with back pain for two years, right sacroiliitis confirmed by scintigraphy and biopsy. She was treated with indomethacin, prednisone, sulfasalazine and metotretaxe showing no significant improvement for one year. Etanercept was initiated with prior normal chest Rx and negative PPD. After 7 months of use, the patient presented fever associated to pleural effusion in the right hemithorax. The biopsy was consistent with pleural tuberculosis. She was treated with rifampicin, isoniazid, pyrazinamide and streptomycin with complete recovery of pulmonary infection.

Case 2: Female patient, 5 years old with juvenile idiopathic arthritis (JIA) polyarticular RF negative. Initially treated with naproxen, methotrexate, prednisone, and folic acid without clinical improvement. After 6 months of treatment, it was initiated infliximab, with prior normal chest Rx and negative PPD with control of the joint symptoms. After 7 months of use, the patient developed severe pneumonic miliary tuberculosis. She was treated with rifampicin, isoniazid, pyrazinamide. The patient resolved her pulmonary infection and remains in remission of JIA, after 7 years of follow-up.

Case 3: Male patient, 17 years old, ankylosing spondylitis for 3 years, treated with methotrexate, naproxen, sulfasalazine and prednisone with poor response. He was started on infliximab for 6 years until he presented productive cough with headache and fever, CXR was consistent with pulmonary tuberculosis. He was treated with rifampicin, isoniazid, pyrazinamide for 6 months. The iTNF was changed for adalimumab with control of the disease.

## Results

Several authors describe that the risk of reactivation of TB increases five to ten times, appearing in a range of up to 1 year after starting iTNF therapy. In case 3, the prolonged time between iTNF administration and the infection suggests that new tuberculosis infection (reinfection) occurred. All patients had normal chest RX and PPD negative at the beginning of the treatment.

## Conclusion

Despite having revolutionized rheumatologic practice, the use of iTNF in the treatment of autoimmune diseases increases the risk of tuberculosis infection and should be carefully monitored especially in countries with high prevalence of TB.

## Disclosure of interest

None declared.

